# Supplementary material for: Stem cell niche exit in C. elegans via orientation and segregation of daughter cells by a cryptic cell outside the niche
Source: eLife. 2020 Jul 21;9:e56383. doi: 10.7554/eLife.56383 (PMC7467730; doi:10.7554/eLife.56383)
Supplement: Supplementary file 1. — Related to Figure 4. [file elife-56383-supp1.docx]

**Supplementary File 1. Time-lapse movies analyzed for interface division asymmetry**

| Dataset | Gonads with DTC divisions | Scored  DTC divisions | Gonads with interface divisions | Scored interface divisions | Asymmetric interface divisions |
| --- | --- | --- | --- | --- | --- |
| tubulin::GFP, germ cell histones, mKate::INX-8, DTC membrane marker | 17 | 26 | 19 | 26 | 22 |
| germ cell histones, germ cell membranes, mKate::INX-8, DTC membrane marker | 9 | 6 | 6 | 11 | 9 |
| ARX-2::GFP, germ cell histones, mKate::INX-8 | 3 | 4 | 10 | 12 | 11 |
| GFP::INX-9, germ cell histones, DTC membrane marker | 12 | 19 | 9 | 15 | 14 |
| **Total** | **41** | **55** | **44** | **64** | **56** |
